# Comparative profiles of BRAF inhibitors: the paradox index as a predictor of clinical toxicity

**DOI:** 10.18632/oncotarget.8351

**Published:** 2016-03-25

**Authors:** Charles H. Adelmann, Grace Ching, Lili Du, Rachael C. Saporito, Varun Bansal, Lindy J. Pence, Roger Liang, Woojin Lee, Kenneth Y. Tsai

**Affiliations:** ^1^ Department of Translational Molecular Pathology, The University of Texas M.D. Anderson Cancer Center, Houston, TX, 77030, USA; ^2^ Department of Dermatology, The University of Texas M.D. Anderson Cancer Center, Houston, TX, 77030, USA; ^3^ Graduate School of Biomedical Sciences, The University of Texas M.D. Anderson Cancer Center, Houston, TX, 77030, USA

**Keywords:** BRAF, squamous cell carcinoma, small molecule inhibitor, paradoxical ERK, melanoma

## Abstract

BRAF inhibitor (BRAFi) therapy is associated with the induction of neoplasia, most commonly cutaneous squamous cell carcinoma (cuSCC). This toxicity is explained in part by “paradoxical ERK activation,” or the hyperactivation of ERK signaling by BRAFi in *BRAF* wild-type cells. However, the rate of cuSCC induction varies widely among BRAFi. To explore this mechanistically, we profiled paradoxical ERK activation by vemurafenib, dabrafenib, encorafenib (LGX818), and PLX8394, demonstrating that vemurafenib induces ERK activation the greatest, while dabrafenib and encorafenib have higher “paradox indices”, defined as the pERK activation EC_80_ divided by the IC_80_ against A375, corresponding to wider therapeutic windows for achieving tumor inhibition without paradoxical ERK activation. Our results identify differences in the paradox indices of these compounds as a potential mechanism for the differences in cuSCC induction rates and highlight the utility of using ERK activity as a biomarker for maximizing the clinical utility of BRAFi.

## INTRODUCTION

In 50% of cutaneous melanomas, activating mutations in *BRAF* drive tumor survival and proliferation through ERK activation [1]. Vemurafenib and dabrafenib were the first selective BRAF inhibitors (BRAFi) approved for clinical use in 2011 and 2013, respectively, and have clinical response rates of about 50% in *BRAF*-mutant melanoma [2]. Newer BRAFi such as encorafenib (LGX818) and PLX8394 have distinct biochemical properties and are still in clinical trials (NCT01909453) [3, 4]; (NCT02012231) [5]. For example, encorafenib is known to have a relatively long off-rate [6], and PLX8394 does not activate ERK in *BRAF*-wild-type cells.

Monotherapy with vemurafenib, dabrafenib, and encorafenib induces neoplasia, most often cutaneous squamous cell carcinoma (cuSCC), at rates of approximately 22%, 6%, and 3.7%, respectively [3, 7–14], averaged across multiple phase I-III trials conducted in heterogeneous patient populations. This effect has been attributed predominantly to “paradoxical ERK activation,” or the ability of BRAFi to stimulate RAF signaling in *BRAF* wild-type contexts, activating ERK and driving oncogenesis [15–19]. Paradoxical ERK activation is most pronounced in *RAS*-mutant cells; accordingly, activating *HRAS* mutations are present in up to 60% of vemurafenib-induced cuSCC [20, 21]. Presumably, *RAS* mutations pre-exist in epidermal keratinocytes prior to BRAFi administration and drug-induced ERK activation drives tumor formation. Though the effects of paradoxical ERK activation most often manifest as cuSCC induction, cases of *NRAS*-mutant leukemia and new primary *BRAF* wild-type melanomas have also been reported [22, 23]. Concomitant inhibition of MEK substantially decreases but does not eliminate cuSCC induction [24, 25]. We have shown that at clinically relevant doses, vemurafenib, but not dabrafenib, potently inhibits JNK signaling and suppresses apoptosis, which cooperates with paradoxical ERK activation to induce tumors [8]. This effect is also seen with the pan-RAF inhibitor sorafenib [26].

While it is clear that BRAFi induce cuSCC with varying efficiency, it is unknown why this is the case, even though the most extensively tested inhibitors, vemurafenib and dabrafenib, appear to have similar efficacy in melanoma [7, 9–14]. We have shown that paradoxical ERK activation accounts for up to 82% of the effect on paradoxical oncogenesis, with the remainder accounted for by off-target inhibition of JNK signaling, which is very prominent with vemurafenib but not dabrafenib [8]. While this might account for the relatively high rate of cuSCC induction with vemurafenib relative to dabrafenib, paradoxical ERK activation has not been directly compared amongst the various BRAFi.

To address this question, we profiled four BRAFi in parallel to explore how patterns of paradoxical ERK activation differed across clinically relevant concentrations. Both the magnitude of peak paradoxical ERK activation and the time course of activation were unique to each inhibitor. We estimated an EC_80_ for inducing ERK activation for each BRAFi, which was then compared to the IC_80_ for growth inhibition of *BRAF*-mutant melanoma cells to derive a paradox index as a means of quantifying a therapeutic window of high clinical efficacy and minimal paradoxical ERK activation. Using these criteria, we identified a potential basis for the clinical observation that BRAFi differ substantially in their ability to induce cuSCC thus highlighting an important potential role for using ERK activity as a biomarker with which to optimize the clinical utility of BRAFi.

## RESULTS

### BRAFi have unique paradoxical ERK activation profiles

**Figure 1:**
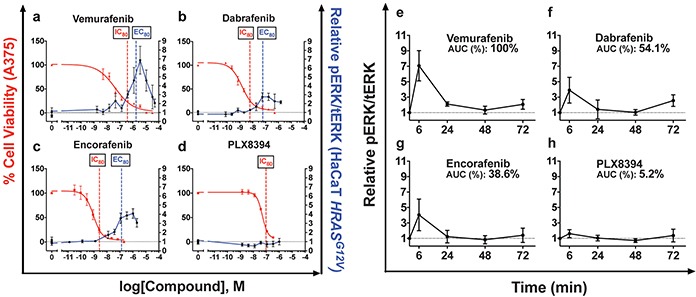
Paradoxical ERK activation profiles and paradox indices correlate with BRAFi clinical toxicities **a–d.** MAPK stimulation in immortalized human keratinocytes (HaCaT) stably expressing *HRAS^G12V^* was measured through quantitative western blotting of phosphorylated ERK (pERK), normalized to total ERK levels after 15 minutes of treatment. Vemurafenib strongly simulated pERK, as did dabrafenib and encorafenib at lower levels. PLX8394 did not stimulate pERK. Efficacy in melanoma was measured with cell viability assay conducted at 72 hours and quantitatively compared to pERK induction in the text. **e–h.** Time-course treatment of HaCaT*^HRASG12V^* at the IC_80_ against A375 of each compound. Treatment was refreshed at 36 hours. AUC measurements rank pERK induction strength from greatest to least: vemurafenib, dabrafenib, encorafenib, PLX8394. Summary parameters for each compound are compiled in Table 1. (*p<0.05; *** p<0.001; *NS =* not significant, n≥3 for all data points).

Four distinct BRAFi were profiled for paradoxical ERK activation across a range of inhibitor doses effective against the *BRAF* mutant melanoma cell line A375 *in vitro*. The immortalized *BRAF* wild-type human keratinocyte cell line HaCaT, stably expressing HRAS^G12V^ [27], was used to model cutaneous epidermal keratinocytes susceptible to paradoxical ERK activation, since this occurs most prominently in the context of mutant *RAS*. HaCaT*^HRASG12V^* cells were exposed to drug for 15 minutes and ERK activation was measured by quantitative western blotting for phosphorylated ERK (pERK) normalized to a total ERK loading control (tERK) (Figure 1a–1d). To compare ERK activation profiles against the effect of melanoma growth inhibition, cell viability at 72 hours with the *BRAF* mutant cell line A375 was assessed in parallel (Figure 1). EC_80_ values for pERK/tERK induction were determined by a four parameter logistic model.

To compare the magnitude of ERK activation to the magnitude of inhibition of *BRAF*-mutant melanoma cells, cell viability of A375 cells following BRAFi treatment was measured in parallel (Figure 1a–1d, Table 1). Potency against A375 was not proportional to pERK induction potency. Therefore, we defined the “paradox index,” an *in vitro* surrogate of a therapeutic index calculated by dividing each pERK induction EC_80_ by the IC_80_ against A375. A greater paradox index indicates a larger window where anti-melanoma activity occurs without activation of ERK. For vemurafenib, the paradox index was narrow, 5.5 (Figure 1a, Table 1), the smallest of all clinically available BRAFi. Dabrafenib had an index of 10 (Figure 1b, Table 1), and encorafenib had the largest at 50 which correlates well with overall rates of cuSCC and perhaps explains how it has comparable cuSCC induction to dabrafenib despite substantially stronger peak pERK induction [3, 4] (Figure 1c, Table 1).

**Table 1:** Summary of comparative toxicity profiles

Compound	Clinical Induction Rate (%)	Melanoma Inhibition log[IC80 in M] ± logSE (antilog in nM)	ERK Induction log[EC80 in M] ± logSE (antilog in nM)	Paradox Index (EC_80_/IC_80_)	Peak ERK induction (Fold-change)^†^	Time-course AUC (% Vem)	Apoptosis Suppression (% DMSO ± SE)^††^	Colony Growth (%DMSO ± SE)^††^
Vemurafenib	22	−6.42 ± 0.24 (380)	−5.58 ± 0.19 (2,100)	5.5	6.86 ± 1.27	100	18.5 ± 2.7***	153.3 ± 8.1*
Dabrafenib	6	−8.23 ± 0.10 (5.9)	−7.23 ± 0.16 (59)	10	2.76 ± 0.34*	54.1	−4.7 ± 2.7*^NS^*	122.0 ± 6.9*
Encorafenib	3.7	−8.59 ± 0.14 (2.6)	−6.89 ± 0.16 (130)	50	4.08 ± 0.16*	38.6	5.2 ± 3.5*^NS^*	121.2 ± 6.3*
PLX8394	—	−7.02 ± 0.06 (94)	—	—	—	5.2	4.0 ± 2.9*^NS^*	103.4 ± 5.7*^NS^*

All statistics were Student's unpaired, two-tailed t-tests. *p≤0.05, ***p<0.001, *NS* = not significant.

† T-test compared compounds to vemurafenib.

†† T-test compared compounds to DMSO control.

Vemurafenib treatment induced pERK more strongly than the other BRAFi, reaching 6.86 ± 1.27 fold above DMSO control at its peak (Figure 1a, Table 1). This was significantly greater than dabrafenib and encorafenib treatment induced peaks, which were 2.76 ± 0.34 and 4.08 ± 0.16 fold higher, respectively, consistent with their lower clinical cuSCC induction rates [7] (Figure 1b–1c, Table 1). Consistent with its design as a paradox breaker, PLX8394 did not stimulate ERK activation (Figure 1d, Table 1) even up to 50 μM (not shown) [5]. It has not been reported whether PLX8394 induces cuSCC in humans [5].

### Time-course profiles reveal short-term peak ERK activation

One unresolved issue was whether paradoxical ERK activation, which occurs quickly following drug administration (Figure 1), is sustained. Multiple feedback loops would be expected to dampen down the initial peak response [28], and very high levels of ERK activation lead to cell cycle arrest [29, 30]. To examine the kinetics of activation profiles and to test whether ERK activation was sustained, we performed a time course experiment spanning 72 hours of drug exposure (Figure 1e–1h). To model paradoxical ERK activation at clinically relevant doses, each inhibitor was tested in parallel at their respective IC_80_ against A375 cells (Table 1). Aside from PLX8394, the general kinetics of ERK activation was similar among of all tested inhibitors, peaking at 6 hours and returning to lower, stable levels by 24 hours (Figure 1e–1h), while recapitulating the magnitude of peak induction in the 15 min ERK profile (Figure 1a–1d).

Peak ERK activation by vemurafenib at 6 hours was 7.07 ± 1.96 fold above control, and was again the highest of all tested inhibitors (Figure 1e). As before, dabrafenib and encorafenib stimulated ERK at lower levels, (Figure 1f–1g), at 3.90 ± 0.34 and 4.04 ± 0.16 fold above control, respectively (Figure 1f–1g, Table 1). As before, PLX8394 did not activate ERK significantly (Figure 1h, Table 1).

To quantify the total time-integrated amount of ERK activation with each inhibitor over 72 hours, we calculated the area under the curve (AUC) for each induction curve in each time course (Figure 1e–1h). Consistent with short-term peak pERK induction at early time points, vemurafenib treatment incurred the highest integrated ERK activation over 72 hours (116.6 hr*pERK/tERK, Table 1). Dabrafenib and encorafenib had smaller total integrated ERK activation profiles as well. The AUC for dabrafenib was 54.1% of that of vemurafenib (63.1 hr*pERK/tERK, Table 1), and the AUC for encorafenib was 38.6% of that of vemurafenib AUC (45.0 hr*pERK/tERK, Table 1). The AUC for PLX8934 was negligible at 5.8 hr*pERK/tERK (Table 1). Overall, ERK activation, as measured by peak activation and the time-integrated AUC, was strongest for vemurafenib. Dabrafenib and encorafenib had lower, and the lowest levels of total ERK activation by these metrics, respectively, among the ATP-competitive BRAFi we tested, correlating again with cuSCC induction rates. ERK activation by PLX8394 was negligible as expected.

### Off-target inhibition of JNK signaling is most relevant for vemurafenib, which has the highest growth-promoting effects

**Figure 2:**
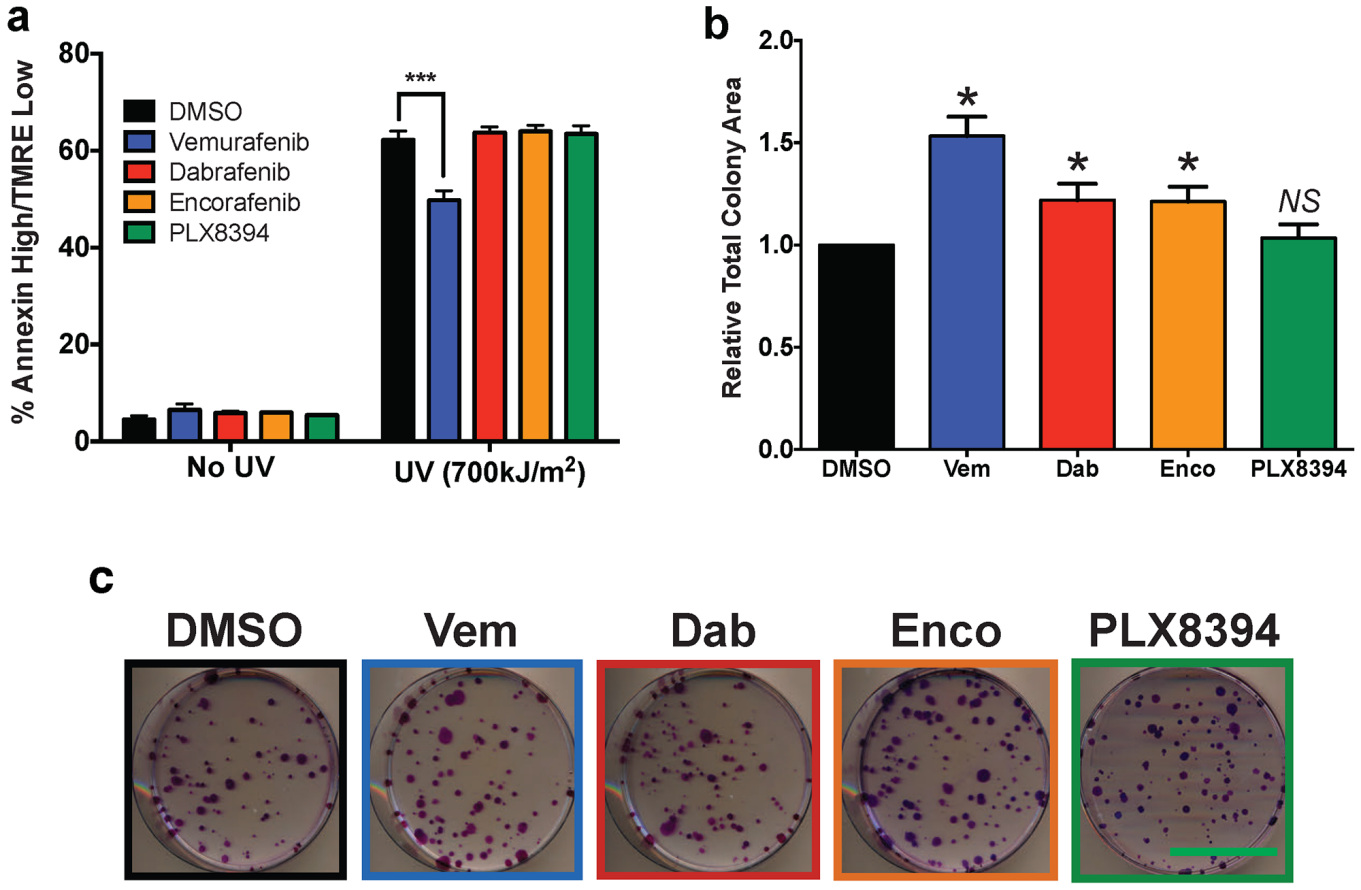
Paradoxical ERK activation and apoptosis suppression profiles are consistent with differences in proliferation **a.** Apoptosis 24 hours after 700J/m*^2^* UV-irradiation of HaCaT*^HRASG12V^* cells measured with AnnexinV/TMRE FACs. Only vemurafenib treatment induced significant apoptosis suppression. **b–c.** Colony growth assay after 12 days of continual drug treatment, quantified by total colony area (ImageJ), showed strong growth advantage for vemurafenib and a detectable advantage with encorafenib and dabrafenib versus control. Scale bar on representative images is 5 cm. All statistical tests were two-tailed, unpaired Student's t-tests. Summary parameters for each compound are compiled in Table 1. (*p<0.05; *** p<0.001; *NS =* not significant, n≥3 for all data points).

Because suppression of JNK-dependent apoptosis is another mechanism that contributes to cuSCC development [8, 26], BRAFi were tested in HaCaT*^HRASG12V^* cells for apoptosis suppression after UV-treatment at their IC_80_ concentrations against A375. Vemurafenib treatment significantly suppressed apoptosis to levels 79.2% of control (Figure 2a, Table 1). No other inhibitor suppressed apoptosis at the IC_80_ against A375 (Figure 2a, Table 1). This suggests that clinically relevant inhibition of melanoma can be achieved without detectable apoptosis suppression, particularly with dabrafenib, encorafenib, and PLX8394. This reinforces conclusions from our paradox profiles that with the exception of vemurafenib, BRAFi dosage can be calibrated to obtain maximal effect against melanoma with minimal toxicity.

To link our findings in paradoxical ERK activation and JNK signaling suppression to cellular proliferation over a sustained period of drug exposure, colony growth assays were performed with each compound at their IC_80_. Consistent with mechanistic observations, vemurafenib treatment strongly increased colony growth 53% above control (Figure 2b, Table 1). Dabrafenib and encorafenib treatment stimulated growth equivalently (21-22%) (Figure 2b, Table 1). As expected, PLX8394 treatment failed to increase growth (Figure 2b, Table 1).

## DISCUSSION

The development of BRAFi has been an unprecedented effort that has revolutionized the treatment of *BRAF*-mutant melanoma. Although the sequence of available BRAF inhibitors has begun to follow a general trend towards lower clinical toxicity and higher potency, no formal side-by-side comparison has been published, particularly with respect to paradoxical ERK activation, a significant driver of common toxicities [8, 15–19]. Furthermore, no mechanistic understanding of why these toxicities differ so significantly between these compounds has been advanced. Here, we studied four BRAFi and generated ERK activation profiles in conjunction with inhibition profiles against *BRAF*-mutant A375 melanoma cells. Two potential explanations for differences in cuSCC induction rates between BRAFi emerged: (1) differing degrees of peak and time-integrated pERK/tERK induction and (2) different “paradox indices.” This measure, the paradox index, is meant to be an *in-vitro* estimation of a therapeutic window, reflecting a concentration range within which efficacy against *BRAF*-mutant melanoma cells is maximized while paradoxical ERK activation is minimized.

It is clear from our study that each BRAFi has distinct clinical toxicity, paradox index (Table 1), and apoptosis suppression profiles (Figure 2a). The defining feature of vemurafenib was strong maximum paradoxical ERK induction that was 1.48-to 2.49-fold higher than other profiled inhibitors (Figure 1, Table 1), an overall effect that was maintained within the first 6 hours of the time course experiment (Figure 1e–1h, Table 1), and in the time-integrated AUC curves (Table 1). Since ERK activation is an important driver of proliferation in BRAFi-induced tumors, substantially stronger ERK induction with vemurafenib is consistent with its greater rate of cuSCC induction as compared to dabrafenib and encorafenib [3, 7–14].

Importantly, dabrafenib and encorafenib both stimulated ERK more modestly by all metrics, but also had substantially higher paradox indices, suggesting that more of each inhibitor can be tolerated before paradoxical ERK activation-driven toxicities would be expected to become evident. Encorafenib had the largest paradox index, which was 5-fold higher than that of dabrafenib, consistent with encorafenib having the lowest reported cuSCC induction rate to date [3]. The wide indices for these two compounds suggest that therapeutic benefit can be optimized against paradoxical ERK activation-driven toxicities clinically, particularly by using ERK activity or pERK/tERK levels as a biomarker.

Interestingly, the profiles of ERK activation all decreased with increasing concentration into the micromolar range (Figure 1). This likely reflects increasing suppression of CRAF activity, which is required for paradoxical ERK activation, and a smaller proportion of RAF dimers in which only one protomer is drug-bound [15–19].

ERK activation for all compounds tested stabilized to lower levels after 72 hours (Figure 1e–1h). Nevertheless, the mechanistic trends inferred from short-term activation profiles, and more importantly clinical cuSCC induction rates, were consistent with AUC measurements when cells were treated at the IC_80_ against A375 cells. Vemurafenib had the largest measured AUC, consistent with the strongest short-term induction of ERK and highest clinical cuSCC rate (Figure 1a, 1e, Table 1) [9–11]. The total paradoxical ERK activation effect, as quantified by the AUC for dabrafenib, was approximately 2-fold lower than the AUC for vemurafenib, correlating with lower peak ERK induction, a wider paradox index, and a lower cuSCC induction rate (Figure 1b, 1f, Table 1) [12–14]. Finally, encorafenib had the lowest AUC of all of the non-paradox breaker BRAFi tested, which was also consistent with its low peak ERK activation, the widest paradox index of the BRAFi tested with detectable paradoxical ERK activation, and the lowest reported cuSCC induction rate (Figure 1c, 1g, Table 1) [3]. Although ERK activation profiles were unique for each BRAFi, paradoxical ERK activation is a general property of this class of compounds, and similar paradoxical effects are seen with other kinase inhibitors, notably for AKT [31, 32].

These profiles are not only consistent with clinical observations, but they highlight a possible mechanism for the differing rates of BRAFi associated toxicities and suggest that ERK activity can be used as a biomarker to optimize the therapeutic window for these drugs. Importantly, these compounds have differing biochemical properties and the differences in paradox indices may reflect differing selectivity and potency against mutant BRAF and CRAF [33, 34]. Differences in *in-vivo* pharmacology and complementary mechanisms such as JNK pathway inhibition exist and vary distinctly between BRAFi [8]. Finally, while our study focused on *RAS* mutant cells, in which paradoxical ERK activation appears to be strongest, this activation occurs in other contexts as well, including EGFR and HER2 overexpression [5].

The number of compounds with overlapping targets and subtle pharmacological differences in the physician's toolbox is expanding. As more complex treatment strategies evolve, such as multiple-combinations and personalized dosing, it will be important to have rigorous comparisons of inhibitor properties before implementing treatment rationally.

## MATERIALS AND METHODS

### Inhibitors

Vemurafenib and dabrafenib were purchased from Selleck Chemicals. PLX8394 was graciously supplied by Chao Zhang, PhD (Plexxikon), and encorafenib by Darrin D. Stuart, PhD (Novartis). All inhibitors were dissolved in DMSO (Sigma). The final DMSO concentration in all experiments described was 0.05% (v/v). All other chemical reagents, if not otherwise specified, were from Fisher.

### Cell culture

HaCaT*^HRASG12V^* were provided by Ulrich Rodeck, PhD (Thomas Jefferson University) and propagated in CellGro DMEM F12 50/50 (Corning) supplemented with 10% HyClone FBS (Invitrogen), CellGro glutamine (Corning), Primocin (Invivogen), and 40μg/ml G418 (Santa Cruz Biotech). A375 was obtained from Suhendan Ekmekcioglu, PhD (MDACC) and were propagated in HyClone RPMI-1640 (Invitrogen) supplemented with 5% HyClone FBS (Invitrogen), CellGro glutamine (Corning), and pen/strep (Corning). All lines were authenticated via the MDACC internal STR profiling service and matched to existing databases.

### Cell viability

A375 cells were plated in culture media into a black-walled 96-well microtiter plate (Corning) overnight. Cells were treated for 72 hours and viability was assessed with Cell Titer Glo (Promega) according to manufacturer's instructions. Sigmoid curve fitting was a four parameter logistic equation implemented with Prism (GraphPad Inc.), fit to normalized values for cell viability. IC_80_ values and associated errors were calculated from the model fit.

### Cell lysates and western blotting

HaCaT*^HRASG12V^* were plated in culture media with 1% FBS (Invitrogen) overnight before drug treatment. Short-term experiments were treated for 15 minutes before lysis. For time course experiments, media and drug were refreshed at 36 hours. Cells were lysed in MPER Extraction Buffer (Thermo) supplemented with Halt Phosphatase and Protease Inhibitor (Thermo). After clearing lysates with centrifugation, protein concentration was normalized with BCA Assay (Thermo), and 15μg total protein/well was loaded into 1.5mm 10% SDS-Polyacrylamide gels after denaturation in standard SDS-loading dye. Running and transfer were in Tris-Glycine buffer (25 mM Tris, 192 mM glycine). Running buffer was supplemented with 0.1% SDS and transfer buffer with 20% ethanol. Transfer was onto a PVDF Immunobilon Membrane (Millipore).

Primary antibodies for western blotting were from Cell Signaling: rabbit phospho-ERK1/2 (D13.14.4E) and mouse total-ERK1/2 (3A7). Secondary antibodies were from Li-Cor: IRDye 680RD goat anti-rabbit IgG and IRDye 800CW goat anti-mouse IgG. Fluorescent western double staining was developed on the Odyssey fluorescent western system (Li-Cor) graciously supplied by Craig D. Logsdon, PhD.

### ERK activation analysis

Band intensity quantification was performed with ImageStudioLite (Li-Cor). pERK and tERK signal was measured only from ERK2, taken to be indicative of both isoforms' activation levels. Each pERK band was normalized to corresponding tERK signal, and pERK/tERK signal was further normalized to DMSO controls run in triplicate. To calculate EC_80_s, a four parameter logistic equation was fit (GraphPad Inc.) to induction curve excluding data points at higher concentrations past peak activation. To accurately model dose response using compounds without defined plateaus, the “top” parameter for every fit was constrained to the peak value listed in Table 1.

Time course band quantification was performed as described above on time course western blots. In addition measuring pERK/tERK intensity at each time point, area under the curve measurements were implemented using the trapezoid method (GraphPad, Inc.).

### Apoptosis assay

UVB irradiation (700 J/m^2^) was administered using an FS40 sunlamp, calibrated with an IL1700 radiometer. Cells were pretreated 1 hour before treatment in PBS, followed by a 24 hour incubation in drug-containing media. Cells were harvested and stained with TMRE (Invitrogen) and Annexin-V-FITC (Invitrogen). Data was collected on a Fortessa (Becton Dickinson) flow cytometer, and analyzed on FlowJo (Tree Start). Apoptotic cells were gated as an ‘Annexin High/TMRE Low’ quadrant.

### Colony formation assay

Cells were plated onto 10 cm dishes at a density of 200 cells/plate. Treatment at the IC_80_ against A375 for each compound was initiated the next day and media was exchanged every 2-3 days for 12 days. Development after methanol fixation was in 0.1% Crystal Violet in 25% Methanol. Plates were digitally scanned and total colony area was quantified in ImageJ (NIH).

## Abbreviations

BRAF: v-Raf murine sarcoma viral oncogene homolog B
ERK: extracellular signal-regulated kinases
cuSCC: cutaneous squamous cell carcinoma

## References

[1] Wan PT, Garnett MJ, Roe SM, Lee S, Niculescu-Duvaz D, Good VM, Jones CM, Marshall CJ, Springer CJ, Barford D, Marais R (2004). Mechanism of activation of the RAF-ERK signaling pathway by oncogenic mutations of B-RAF. Cell.

[2] Jang S, Atkins MB (2014). Treatment of BRAF-mutant melanoma: the role of vemurafenib and other therapies. Clin Pharmacol Ther.

[3] Dummer R, Robert C, Nyakas M, McArthur G, Kudchadkar R, Gomez Roca C, Sullivan R, Flaherty K, Murer C, Michel D, Tang Z, Moutouh-de Parseval L, Delord JP (2013). Initial results from a phase I, open-label, dose escalation study of the oral BRAF inhibitor LGX818 in patients with BRAF V600 mutant advanced or metastatic melanoma. J Clin Oncol.

[4] Sullivan R, Weber J, Patel S, Dummer R, Miller WH, Cosgrove D, Carlino M, Tan D, Lebbe C, Cipani T, Elez E, Maacke H, Nikolopoulos P, Aout M, Moutouh-de Parseval L, Ascierto P (2013). A phase Ib/II study of BRAF inhibitor (BRAFi) encorafenib (ENCO) plus MEK inhibitor (MEKi) binimetinib (BINI) in cutaneous melanoma patients naive to BRAFi treatment. J Clin Oncol.

[5] Zhang C, Spevak W, Zhang Y, Burton EA, Ma Y, Habets G, Zhang J, Lin J, Ewing T, Matusow B, Tsang G, Marimuthu A, Cho H, Wu G, Wang W, Fong D (2015). RAF inhibitors that evade paradoxical MAPK pathway activation. Nature.

[6] Stuart DD, Li N, Poon DJ, Aardalen K, Kaufman S, Merritt H, Salangsang F, Lorenzana E, Li A, Ghoddusi M, Caponigro G, Sun F, Kulkarni S, Kakar S, Turner N, Zang R (2012). Abstract 3790: Preclinical profile of LGX818: A potent and selective RAF kinase inhibitor. Cancer Research.

[7] Menzies AM, Kefford RF, Long GV (2013). Paradoxical oncogenesis: are all BRAF inhibitors equal?. Pigment Cell Melanoma Res.

[8] Vin H, Ojeda SS, Ching G, Leung ML, Chitsazzadeh V, Dwyer DW, Adelmann CH, Restrepo M, Richards KN, Stewart LR, Du L, Ferguson SB, Chakravarti D, Ehrenreiter K, Baccarini M, Ruggieri R (2013). BRAF inhibitors suppress apoptosis through off-target inhibition of JNK signaling. Elife.

[9] Sosman JA, Kim KB, Schuchter L, Gonzalez R, Pavlick AC, Weber JS, McArthur GA, Hutson TE, Moschos SJ, Flaherty KT, Hersey P, Kefford R, Lawrence D, Puzanov I, Lewis KD, Amaravadi RK (2012). Survival in BRAF V600-mutant advanced melanoma treated with vemurafenib. N Engl J Med.

[10] Chapman PB, Hauschild A, Robert C, Haanen JB, Ascierto P, Larkin J, Dummer R, Garbe C, Testori A, Maio M, Hogg D, Lorigan P, Lebbe C, Jouary T, Schadendorf D, Ribas A (2011). Improved survival with vemurafenib in melanoma with BRAF V600E mutation. N Engl J Med.

[11] Flaherty KT, Puzanov I, Kim KB, Ribas A, McArthur GA, Sosman JA, O'Dwyer PJ, Lee RJ, Grippo JF, Nolop K, Chapman PB (2010). Inhibition of mutated, activated BRAF in metastatic melanoma. N Engl J Med.

[12] Falchook GS, Long GV, Kurzrock R, Kim KB, Arkenau TH, Brown MP, Hamid O, Infante JR, Millward M, Pavlick AC, O'Day SJ, Blackman SC, Curtis CM, Lebowitz P, Ma B, Ouellet D (2012). Dabrafenib in patients with melanoma, untreated brain metastases, and other solid tumours: a phase 1 dose-escalation trial. Lancet.

[13] Long GV, Trefzer U, Davies MA, Kefford RF, Ascierto PA, Chapman PB, Puzanov I, Hauschild A, Robert C, Algazi A, Mortier L, Tawbi H, Wilhelm T, Zimmer L, Switzky J, Swann S (2012). Dabrafenib in patients with Val600Glu or Val600Lys BRAF-mutant melanoma metastatic to the brain (BREAK-MB): a multicentre, open-label, phase 2 trial. Lancet Oncol.

[14] Hauschild A, Grob JJ, Demidov LV, Jouary T, Gutzmer R, Millward M, Rutkowski P, Blank CU, Miller WH, Kaempgen E, Martin-Algarra S, Karaszewska B, Mauch C, Chiarion-Sileni V, Martin AM, Swann S (2012). Dabrafenib in BRAF-mutated metastatic melanoma: a multicentre, open-label, phase 3 randomised controlled trial. Lancet.

[15] Hatzivassiliou G, Song K, Yen I, Brandhuber BJ, Anderson DJ, Alvarado R, Ludlam MJ, Stokoe D, Gloor SL, Vigers G, Morales T, Aliagas I, Liu B, Sideris S, Hoeflich KP, Jaiswal BS (2010). RAF inhibitors prime wild-type RAF to activate the MAPK pathway and enhance growth. Nature.

[16] Poulikakos PI, Zhang C, Bollag G, Shokat KM, Rosen N (2010). RAF inhibitors transactivate RAF dimers and ERK signalling in cells with wild-type BRAF. Nature.

[17] Heidorn SJ, Milagre C, Whittaker S, Nourry A, Niculescu-Duvas I, Dhomen N, Hussain J, Reis-Filho JS, Springer CJ, Pritchard C, Marais R (2010). Kinase-dead BRAF and oncogenic RAS cooperate to drive tumor progression through CRAF. Cell.

[18] Halaban R, Zhang W, Bacchiocchi A, Cheng E, Parisi F, Ariyan S, Krauthammer M, McCusker JP, Kluger Y, Sznol M (2010). PLX4032, a selective BRAF(V600E) kinase inhibitor, activates the ERK pathway and enhances cell migration and proliferation of BRAF melanoma cells. Pigment Cell Melanoma Res.

[19] Karreth FA, DeNicola GM, Winter SP, Tuveson DA (2009). C-Raf inhibits MAPK activation and transformation by B-Raf(V600E). Mol Cell.

[20] Oberholzer PA, Kee D, Dziunycz P, Sucker A, Kamsukom N, Jones R, Roden C, Chalk CJ, Ardlie K, Palescandolo E, Piris A, Macconaill LE, Robert C, Hofbauer GF, McArthur GA, Schadendorf D (2012). RAS Mutations Are Associated With the Development of Cutaneous Squamous Cell Tumors in Patients Treated With RAF Inhibitors. J Clin Oncol.

[21] Su F, Viros A, Milagre C, Trunzer K, Bollag G, Spleiss O, Reis-Filho JS, Kong X, Koya RC, Flaherty KT, Chapman PB, Kim MJ, Hayward R, Martin M, Yang H, Wang Q (2012). RAS mutations in cutaneous squamous-cell carcinomas in patients treated with BRAF inhibitors. N Engl J Med.

[22] Callahan MK, Rampal R, Harding JJ, Klimek VM, Chung YR, Merghoub T, Wolchok JD, Solit DB, Rosen N, Abdel-Wahab O, Levine RL, Chapman PB (2012). Progression of RAS-mutant leukemia during RAF inhibitor treatment. N Engl J Med.

[23] Zimmer L, Hillen U, Livingstone E, Lacouture ME, Busam K, Carvajal RD, Egberts F, Hauschild A, Kashani-Sabet M, Goldinger SM, Dummer R, Long GV, McArthur G, Scherag A, Sucker A, Schadendorf D (2012). Atypical melanocytic proliferations and new primary melanomas in patients with advanced melanoma undergoing selective BRAF inhibition. J Clin Oncol.

[24] Jang S, Atkins MB (2013). Which drug, and when, for patients with BRAF-mutant melanoma?. Lancet Oncol.

[25] Flaherty KT, Infante JR, Daud A, Gonzalez R, Kefford RF, Sosman J, Hamid O, Schuchter L, Cebon J, Ibrahim N, Kudchadkar R, Burris HA, Falchook G, Algazi A, Lewis K, Long GV (2012). Combined BRAF and MEK Inhibition in Melanoma with BRAF V600 Mutations. N Engl J Med.

[26] Vin H, Ching G, Ojeda SS, Adelmann CH, Chitsazzadeh V, Dwyer DW, Ma H, Ehrenreiter K, Baccarini M, Ruggieri R, Curry JL, Ciurea AM, Duvic M, Busaidy NL, Tannir NM, Tsai KY (2014). Sorafenib Suppresses JNK-Dependent Apoptosis through Inhibition of ZAK. Mol Cancer Ther.

[27] Boukamp P, Stanbridge EJ, Foo DY, Cerutti PA, Fusenig NE (1990). c-Ha-ras oncogene expression in immortalized human keratinocytes (HaCaT) alters growth potential in vivo but lacks correlation with malignancy. Cancer Res.

[28] Lito P, Pratilas CA, Joseph EW, Tadi M, Halilovic E, Zubrowski M, Huang A, Wong WL, Callahan MK, Merghoub T, Wolchok JD, de Stanchina E, Chandarlapaty S, Poulikakos PI, Fagin JA, Rosen N (2012). Relief of profound feedback inhibition of mitogenic signaling by RAF inhibitors attenuates their activity in BRAFV600E melanomas. Cancer Cell.

[29] Das Thakur M, Salangsang F, Landman AS, Sellers WR, Pryer NK, Levesque MP, Dummer R, McMahon M, Stuart DD (2013). Modelling vemurafenib resistance in melanoma reveals a strategy to forestall drug resistance. Nature.

[30] Dimauro T, David G (2010). Ras-induced senescence and its physiological relevance in cancer. Curr Cancer Drug Targets.

[31] Han EK, Leverson JD, McGonigal T, Shah OJ, Woods KW, Hunter T, Giranda VL, Luo Y (2007). Akt inhibitor A-443654 induces rapid Akt Ser-473 phosphorylation independent of mTORC1 inhibition. Oncogene.

[32] Chan TO, Zhang J, Rodeck U, Pascal JM, Armen RS, Spring M, Dumitru CD, Myers V, Li X, Cheung JY, Feldman AM (2011). Resistance of Akt kinases to dephosphorylation through ATP-dependent conformational plasticity. Proc Natl Acad Sci U S A.

[33] Laquerre S, Arnone M, Moss K, Yang J, Fisher K, Kane-Carson LS, Smitheman K, Ward J, Heidrich B, Rheault T, Adjabeng G, Hornberger K, Stellwagen J, Waterson A, Han C, Mook RA (2009). Abstract B88: A selective Raf kinase inhibitor induces cell death and tumor regression of human cancer cell lines encoding B-RafV600E mutation. Molecular Cancer Therapeutics.

[34] Bollag G, Hirth P, Tsai J, Zhang J, Ibrahim PN, Cho H, Spevak W, Zhang C, Zhang Y, Habets G, Burton EA, Wong B, Tsang G, West BL, Powell B, Shellooe R (2010). Clinical efficacy of a RAF inhibitor needs broad target blockade in BRAF-mutant melanoma. Nature.

